# Characterization of semi-polar (20$\overline{2}$1) InGaN microLEDs

**DOI:** 10.1038/s41598-020-72720-1

**Published:** 2020-09-29

**Authors:** Ray-Hua Horng, Shreekant Sinha, Yuh-Renn Wu, Fu-Guo Tarntair, Jung Han, Dong-Sing Wuu

**Affiliations:** 1 Institute of Electronics, National Chiao Tung University, Hsinchu, 30010 Taiwan, ROC; 2 Center for Emergent Functional Matter Science, National Chiao Tung University, Hsinchu, 30010 Taiwan, ROC; 3 Department of Photonics, National Chiao Tung University, Hsinchu, 30010 Taiwan, ROC; 4 Graduate Institute of Photonics and Optoelectronics, National Taiwan University, Taipei, 10617 Taiwan, ROC; 5 Department of Electrical Engineering, Yale University, New Haven, CT 06520 USA; 6 Department of Materials Science and Engineering, National Chung Hsing University, Taichung, 40227 Taiwan, ROC

**Keywords:** Engineering, Materials science, Optics and photonics, Physics

## Abstract

In this paper, semi-polar (20$\overline{2}$1) InGaN blue light-emitting diodes (LEDs) were fabricated and compared the performance with those of LEDs grown on c-plane sapphire substrate. LEDs with different chip sizes of 100 μm × 100 μm, 75 μm × 75 μm, 25 μm × 25 μm, and 10 μm × 10 μm were used to study the influence of chip size on the device performance. It was found that the contact behavior between the n electrode and the n-GaN layer for the semi-polar (20$\overline{2}$1) LEDs was different from that for the LEDs grown on the c-plane device. Concerning the device performance, the smaller LEDs provided a larger current density under the same voltage and presented a smaller forward voltage. However, the sidewall’s larger surface to volume ratio could affect the IQE. Therefore, the output power density reached the maximum with the 25 μm × 25 μm chip case. In addition, the low blue-shift phenomenon of semi-polar (20$\overline{2}$1) LEDs was obtained. The larger devices exhibited the maximum IQE at a lower current density than the smaller devices, and the IQE had a larger droop as the current density increased for the LEDs grown on c-plane sapphire substrate.

## Introduction

It is well known that there are many advantages of solid state light emitting diodes (LEDs), such as a small size, long life, short response time, low power consumption, no need for cooling time after being turned off, et al*.*^1^*.* Because LEDs have these advantages, they are used in a wide range of applications, such as displays, indicators, automotive uses, lighting, electronic equipment, and biomedical applications. It is worthy to mention that LEDs are self-emitting light sources, meaning they can be applied not only to backlights but also to self-emitting displays. Moreover, they can be fabricated to have more pixels per inch (PPI) than organic LEDs with a dimension of LEDs of less than 100 μm, which are known as micro-LEDs (μ-LEDs), and which causes μ-LEDs to be more competitive.

On the other hand, InGaN-based μ-LEDs, which can be applied to various displays, have become widely popular and have developed rapidly in recent years. The advantages of μ-LEDs are the characteristics of high efficiency, high brightness, high reliability, fast response times, self-lighting without a backlight, energy-saving, and a small size^2^. Especially, μ-blue LEDs are not only applied in displays but also in undersea submarine communication, pico-projectors, and surgical devices, etc^3–5^. For these applications, wavelength stability is the main criteria for color tuning. In this work, the leakage current characteristic due to shrinking the device size and its effect on electrical and optical characteristics were evaluated. However, small-sized LEDs with dimensions less than 50 μm cannot be successfully probed with traditional measurement systems. In order to solve this problem, special electrodes for smaller devices were designed and facilitated for measurement using traditional measurement equipment. Moreover, most of optoelectronic InGaN-based devices were grown on a c-plane sapphire substrate (CPSS). Due to the polarized electric field, the electron–hole recombination efficiency of the devices was poor and exhibited the blue shift phenomenon. To address these problems, μ-blue LED epitaxial layers with semi-polar (20$\overline{2}$1) orientation were also grown on a (22$\overline{4}$3) pattern sapphire substrate (SPSS) and fabricated for comparison.

## Results and disscusion

**Figure 1:**
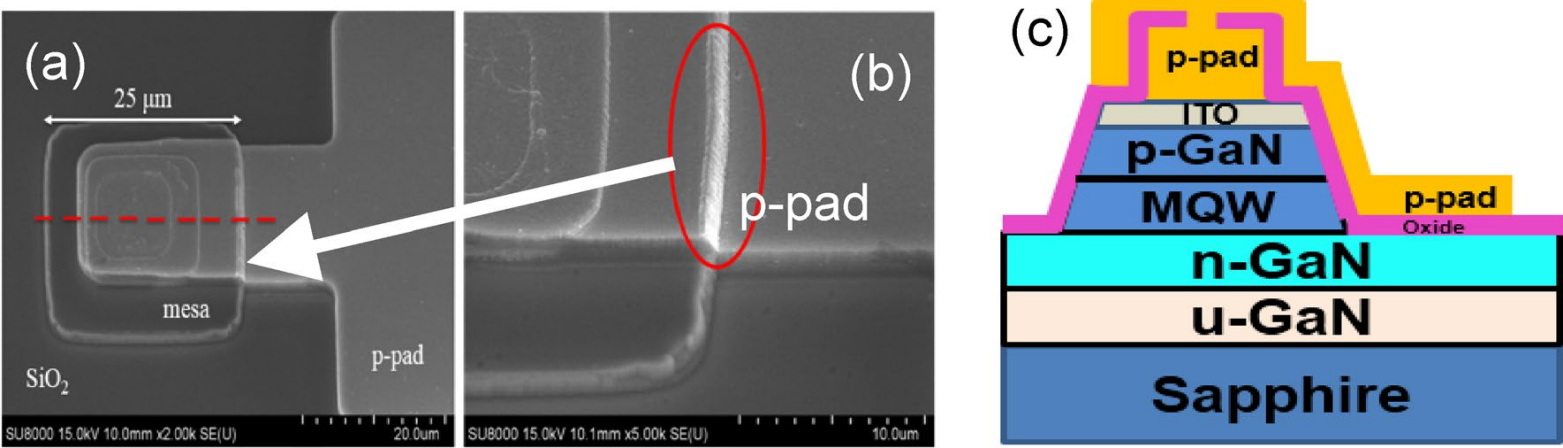
(**a**) SEM image of metal electrode plated on the side wall, (**b**) schematic diagram of the cross section and (**c**) schematic structural view of the red dotted line shown in Fig. 1a.

The LEDs created on these two blue epitaxial structures had four different sizes: 100 μm × 100 μm, 75 μm × 75 μm, 25 μm × 25 μm, and 10 μm × 10 μm, which were all used to evaluate the influence of chip size and optical characteristics. In this study, the above LEDs were defined as LED(100), LED(75), LED(25), and LED(10), respectively. Four different chip sizes (LED(100), LED(75), LED(25), and LED(10)) of blue LEDs with CPSS and SPSS orientations were fabricated. Among them, the electrode size of the LED(25) and LED(10) devices were too small to measure with the probe. In order to measure the optoelectronic properties of the μ-LEDs, an extra p-contact electrode was designed. Figure 1a shows the SEM image of a typical LED(25). A large metal area was successfully deposited on the sidewall to facilitate the p-metal pad. Figure 1c shows the schematic structural view of the red dotted line in Fig. 1a. It was important to obtain the sidewall metal deposition of the light-emitting area, using the slope shown in Fig. 1b, by the dry etching mesa process. Therefore, SiO_2_ could be deposited on the sidewall as the passivation layer, and the deposited metal electrode could be easily extended to the electrode area without disconnection.

**Figure 2:**
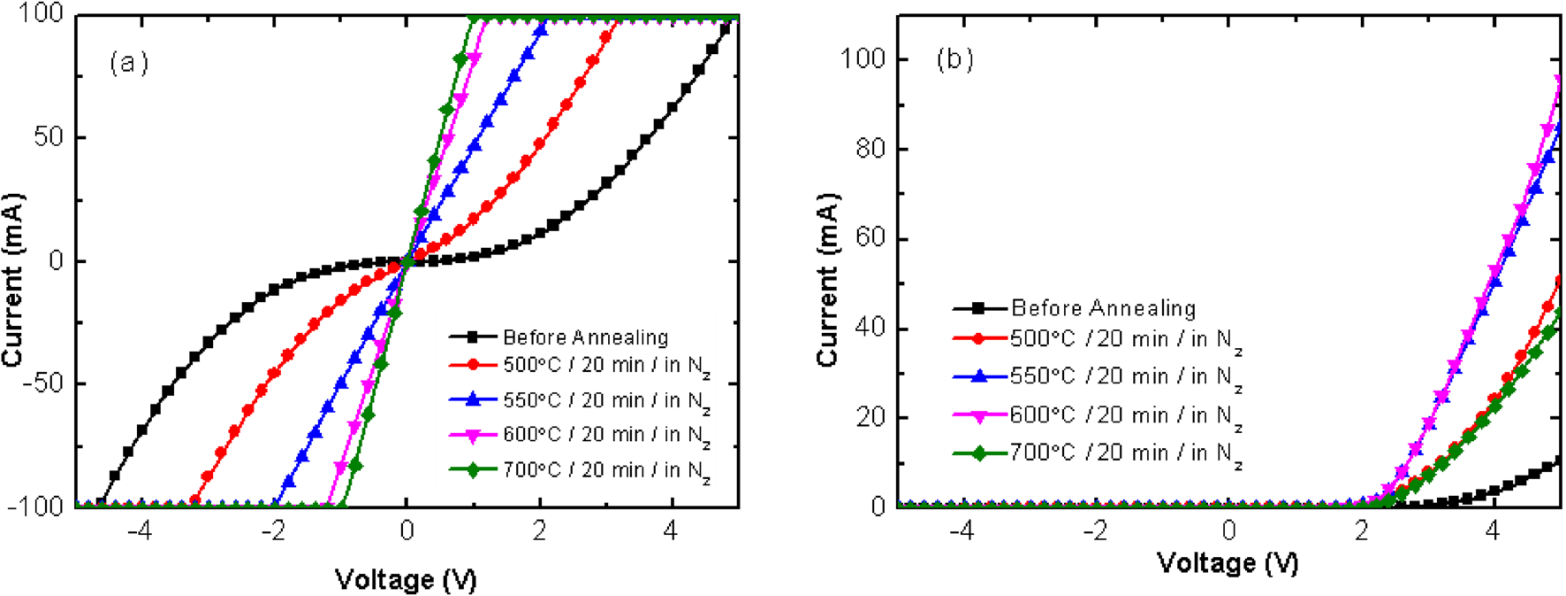
(**a**) I–V curves present the contact properties between the n-electrode and the n-GaN layer before and after annealing process at different conditions and (**b**) I–V characteristics of LEDs before and after annealing processes at different conditions.

In general, there were no additional processes for the Ohmic contact behavior between the n-metal and n-type GaN epitaxial layers for the CPSS LED. However, the deposited n-metal on the n-type GaN epitaxial layer for the SPSS LED did not present the Ohmic contact behavior shown in Fig. 2a. The n-electrode and n-type GaN of SPSS LED did not get an Ohmic contact at an annealing temperature of 500 °C for 20 min. As the annealing temperature neared 550 °C, the contact barrier disappeared between the contact metal and the n-type GaN, even at a high resistance of about 20 Ω. In order to improve the contact resistance, the annealing temperature was increased. A higher annealing temperature could improve the contact characteristic between the n electrode and the n-GaN. But the device becomes worst when an annealing temperature treated at 700 °C, as shown in Fig. 2b. It could be because the 700 °C temperature was too high, which affected the contact characteristic between the ITO layer and the p-GaN. The contact characteristic between the n-electrode and the n-GaN was improved, but the electrical properties of the entire device deteriorated, as shown in Fig. 2b. Based on the above measurement results, the optimum annealing parameters for the n-type contact layer processing of SPSS LEDs were 600 °C with nitrogen atmosphere for 20 min.

**Figure 3:**
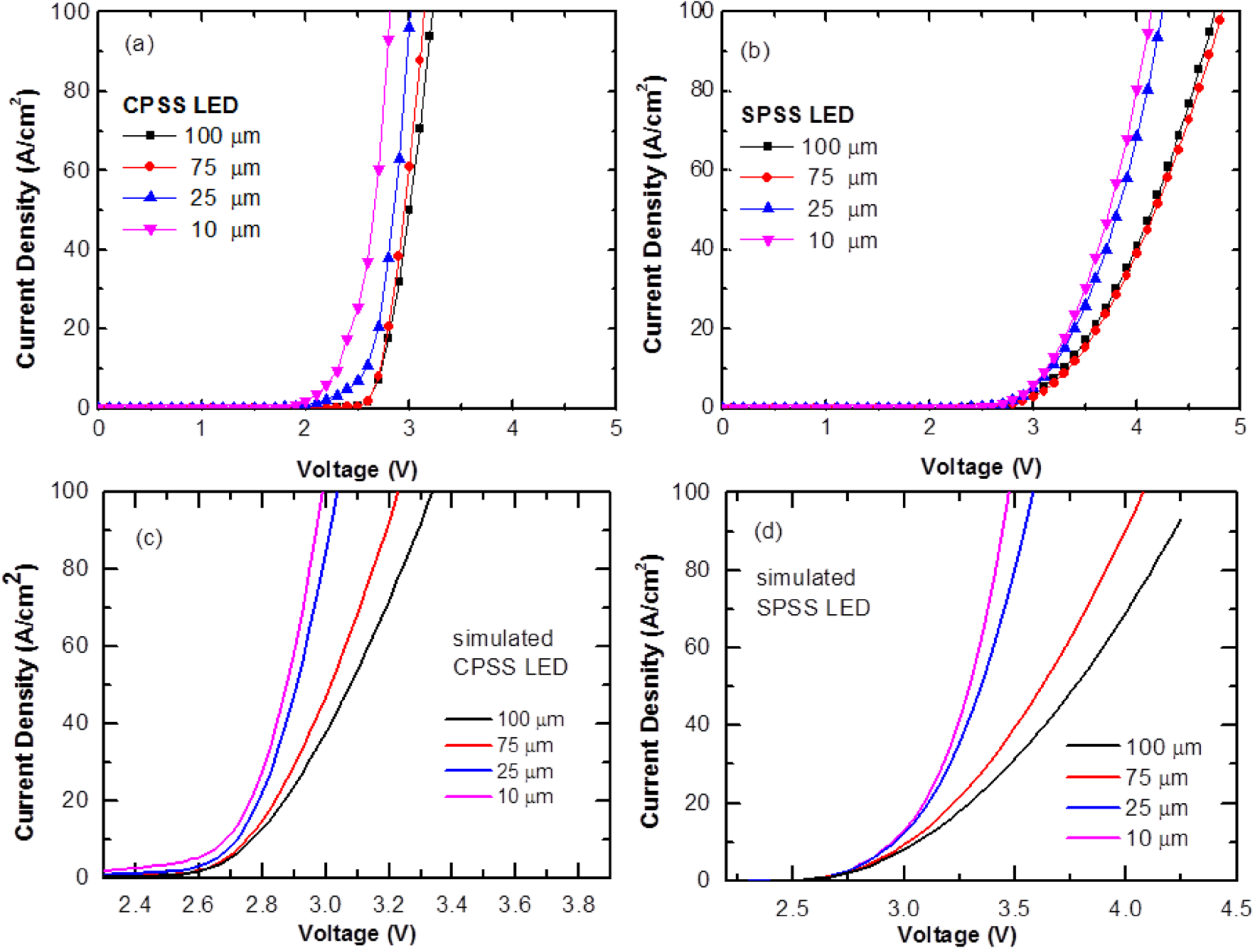
(**a**) J–V characteristics of CPSS blue LEDs with different chip sizes. (**b**) J–V characteristics of SPSS blue LEDs with different chip sizes. (**c**,**d**) are simulated J–V characteristics of CPSS and SPSS LEDs, respectively.

Figure 3a,b show the current density versus the voltage (J–V) of the CPSS and SPSS LEDs using different chip sizes, respectively. It was found that the electrical properties of the LEDs were correlated with the different sizes. The forward voltage increased with the increasing chip size and the smaller LEDs provided a larger current density at the same voltage, except for the 75 $\mathrm{\mu m}$ chip in the semipolar plane case. The voltage at current density equal to 100 A/cm^2^ of LED(100), LED(75), LED(25), and LED(10) on CPSS were 3.23 V, 3.14 V, 3.01 V, and 2.82 V, respectively. In contrast, the voltage of LED(100), LED(75), LED(25), and LED(10) on SPSS at 100 A/cm^2^ were approximately 4.75 V, 4.83 V, 4.24 V, and 4.13 V, respectively. Even the volatge of LED(75) was a little higher than that of LED(100) for the SPSS, the tendency of both LEDs were very similar.

**Figure 4:**
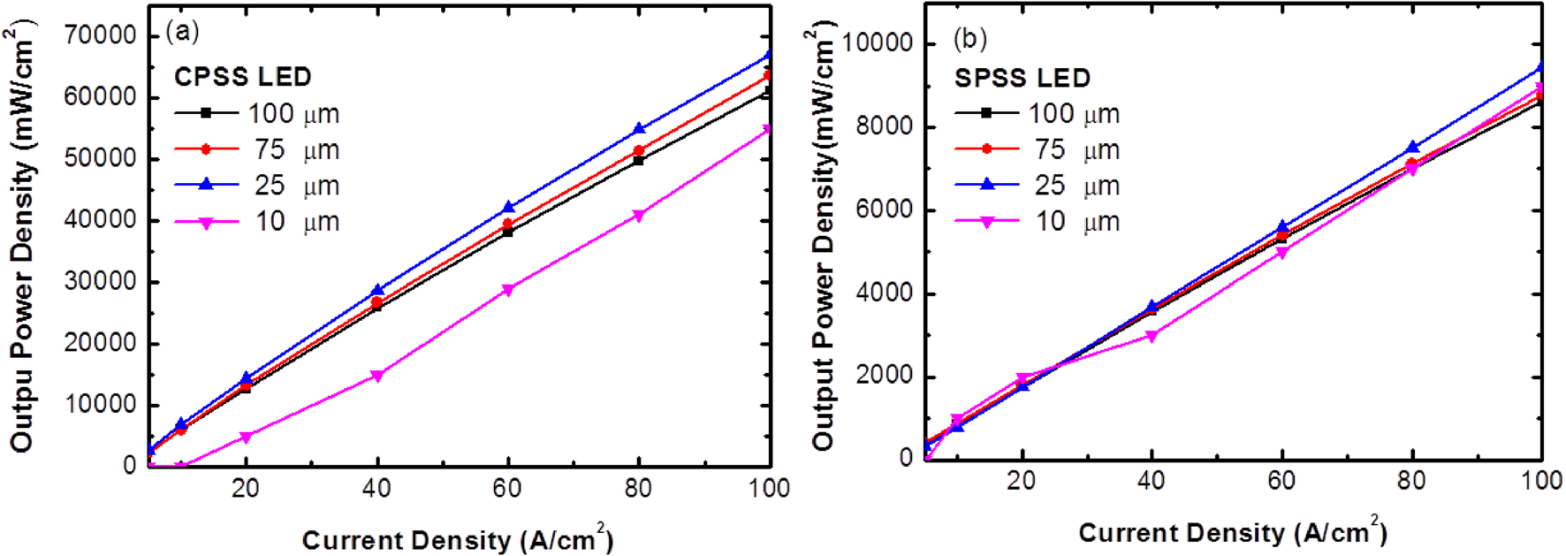
(**a**,**b**) are output power density versus current density for different chip sizes for CPSS LEDs and SPSS LEDs, respectively.

The output power density (as a function of the current density) from 5 to 100 A/cm^2^ was measured by the integrating sphere to understand the influence of LEDs with different chip sizes with the CPSS and SPSS. Figure 4 shows the characteristic of the output power density as a function of the current density for the LEDs with the CPSS and SPSS using different chip sizes. The output power density increased as the chip size decreased^6^ for the CPSS LEDs. Nevertheless, the output power density of LED(10) with the CPSS was the lowest among these chips. On the other hand, the output power density as a function of the current density was almost the same for the SPSS LEDs with different sizes. However, the output of the SPSS LED was much smaller compared to the CPSS LED cases. To explain these trends, it was necessary to first look at the ideality factor:

1 $$n_{ideality} = \frac{q}{{{\text{kT}}}} \times \left( {\frac{\partial lnI}{{\partial V}}} \right)^{ - 1}$$

**Figure 5:**
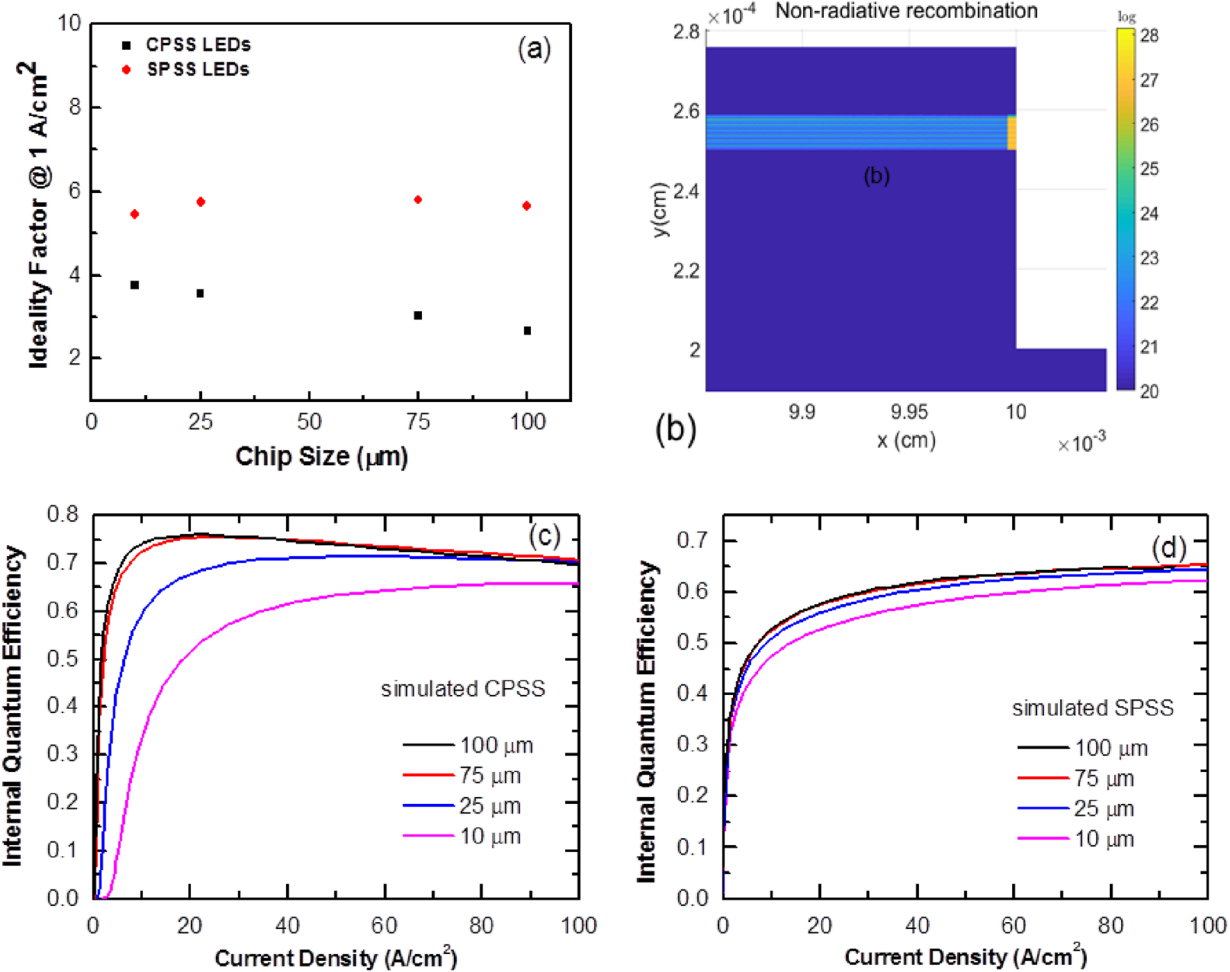
(**a**) Ideality factor of c-plane and semi-polar blue LEDs at 1 A/cm^2^ as a function of chip sizes. (**b**) nonradaitve recombination distribution of c-plane micro LED near the sidewall edge. (**c**) The calculated IQE curve of the c-plane QW LED. (**d**) The calculated IQE curve of the semipolar-plane QW LED.

Using Eq. (1), Fig. 5a illustrates the ideality factor of the CPSS and SPSS LEDs at a current density of 1 A/cm^2^ as a function of the chip size. In Eq. (1), q is the elementary charge, k is the Boltzmann constant, and T is the absolute temperature^7–9^. The ideality factor gradually decreased from 3.75 to 2.66 as the chip size increased from 10 to 100 μm for the CPSS LEDs. On the other hand, the ideality factor varied from 5.45 to 5.65 as the chip size increased from 10 to 100 μm for the SPSS LEDs. An ideality factor of 1 indicated a band to band radiative recombination^10^; when the ideality factor was greater than 2, it indicated defect-assisted tunneling or the nonradiative recombination phenomenon^7, 11^.

The experimental results showed that the CPSS data had a smaller ideality factor at the low current density region compared to the SPSS data. This could be explained by the better output power density of CPSS compared to that of SPSS, as shown in Fig. 4. As the CPSS samples had a mature and well-developed epi-structure, the defect-assisted tunneling phenomenon should have been better compared to CPSS^12^. However, a comparison of the ideality factor in the CPSS data with different chip sizes found that the smaller chip sizes had relatively larger ideality factors. This may have been due to the influence of sidewall surface defects^13^. To explain the influence of sidewall, a simulation was performed to examine the reason for the high ideality factor. The simulation used a tail state model near the band edge in the active region to model the influence of defects leakage. Since the nonradiative recombination at the sidewall surface state is an important issue affecting IQE, we put a higher density of tail states on the sidewall with a much shorter nonradiative lifetime (0.1 ns) to model the efficiency in the chips with a small size. Figure 3c,d show the modeled J–V curves for the LEDs grown on CPSS and SPSS, respectively. For the CPSS LEDs, the simulation showed that near the turn-on voltage, the current flows through sidewall first where most current recombined nonradiatively at low current density region. Of the four different chip sizes, the smallest chip had a larger surface to active volume ratio. Therefore, a larger current density could be seen near the turn-on voltage, as it had the most current flowing through the sidewall surface states. A check of the experimental result at the same voltage showed that the current density was inversely proportional to the chip width. Note that the simulation was based on a 2D structure due to computational time limits, thus it could only model two sidewalls on the 2D plane instead of four sidewalls. Therefore, for the 10 $\upmu$m chip size, the actual sidewall to volume ratio would be much larger in real 3D cases. The simulation might underestimate the sidewall current through the surface state in the smaller chips At a larger bias, the electrons started to flow through the normal chip area^14^.

Figure 5c shows the simulated IQE curve for the CPSS LEDs. It could be found that the IQE of the 10 $\mathrm{\mu m}$ LED chip was lower due to the sidewall nonradiative recombination through defects, as shown in Fig. 5b. Therefore, for a current density smaller than 10 A/cm^2^, most currents were nonradiatively recombined at the sidewall^15, 16^. This result was confirmed with the experimental result, as shown in Fig. 4a, where the 10 $\mathrm{\mu m}$ chip had a lower output power density. Nevertheless, the simulated IQE of LED(100) was the highest among these four chips. However, the output power also relied on the light extraction efficiency (LEE). The smaller chip sizes could usually provide a better LEE due to having a shorter escaping path^17–21^. For the SPSS LED cases, as the IQE of the SPSS LEDs was much worse compared to the CPSS LED cases, the nonradiative lifetime was also short in the bulk active region due to the unoptimized epi-layer. Therefore, the relative influences from the sidewall were weaker compared to the CPSS LEDs, and there was little difference in the IQE for different chip sizes, as shown in Fig. 5d.

**Figure 6:**
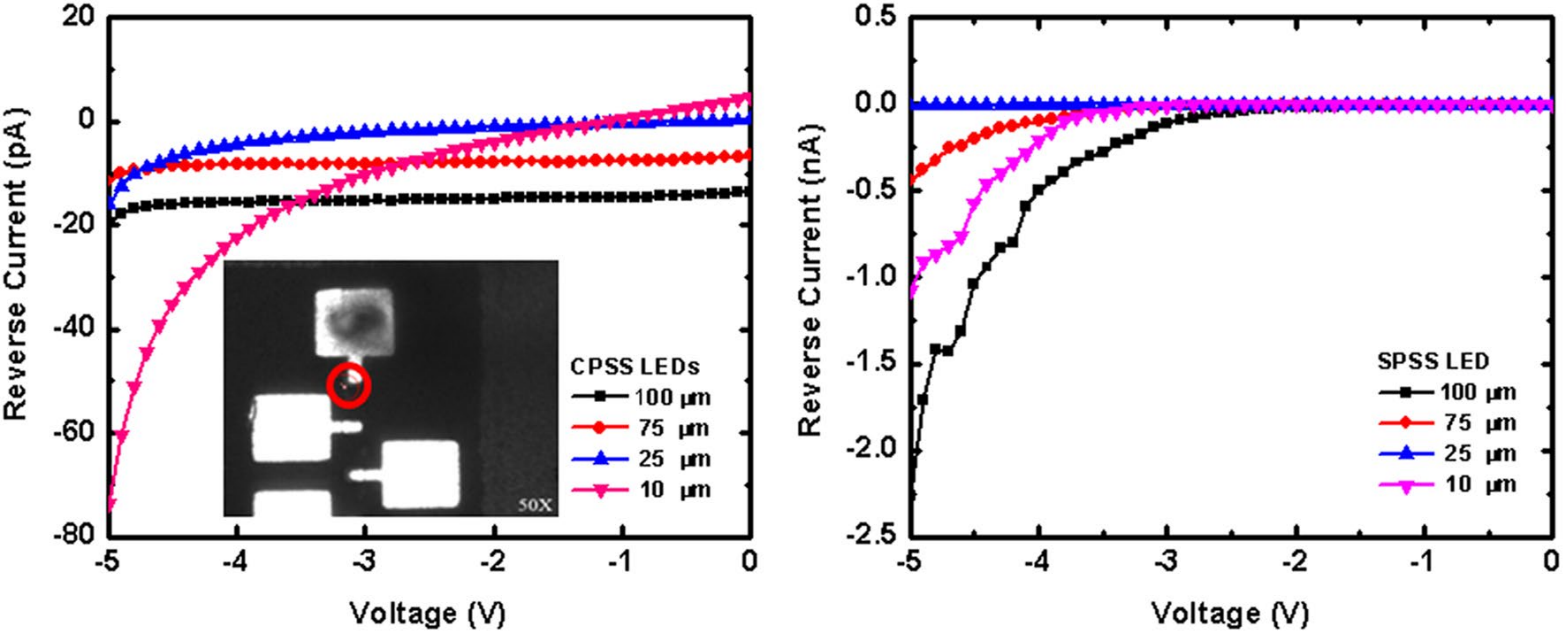
(**a**) Reverse bias I–V characteristics of CPSS LEDs. (**b**) Reverse bias I–V characteristics of SPSS LEDs. Inset is the image measured by InGaAs EMMI.

As the size of the LED was reduced, the ratio between the area and the edge became smaller. Therefore, it was necessary to evaluate whether the reverse leakage current characteristic of the device would become serious due to the reduction in size. Figure 6a,b show the reverse I–V characteristic for four different chip sizes of the blue LEDs with CPSS and SPSS, respectively. The leakage current (@− 5 V) of blue LED(100), LED(75), LED(25), and LED(10) with CPSS were about − 19.7 pA, − 11.5 pA, − 15.9 pA, and − 73.7 pA, respectively, and the leakage current of blue LED(100), LED(75), LED(25), and LED(10) with SPSS were about − 2.3 nA, − 0.4 nA, − 13.3 pA, and − 1.1 nA, respectively. There was no large reversed leakage current in the small-size LEDs if the passivation step was included in the process; therefore, the leakage current could be effectively reduced. Afterwards, this study performed emission microscopy (EMMI) on the c-plane using a 10 μm blue LED to further understand the possible leakage path. InGaAs EMMI as a sensor was used and found to be suitable for advance processing to find the bright spots indicating possible leakage. The principle of EMMI is to use a microscope and a photo-detector to detect the photons excited by the electron–hole combination and the hot carriers. The detectable wavelength range of 900–1700 nm was relatively high and in the infrared band. The operating voltage and the energy of the hot carriers were decreased. The inset of Fig. 6a shows the measurement of the leakage current for CPSS LED(10) by InGaAs EMMI. No bright spot was observed when the device was operated at a reverse bias from − 5 to − 15 V, but a bright spot was observed at the corner of the device when it was applied to − 20 V. Since μ-LEDs do not to be operated at such high voltages during normal application, this result indicated that there would be no leakage issue on the device if the sidewall was properly protected.

**Figure 7:**
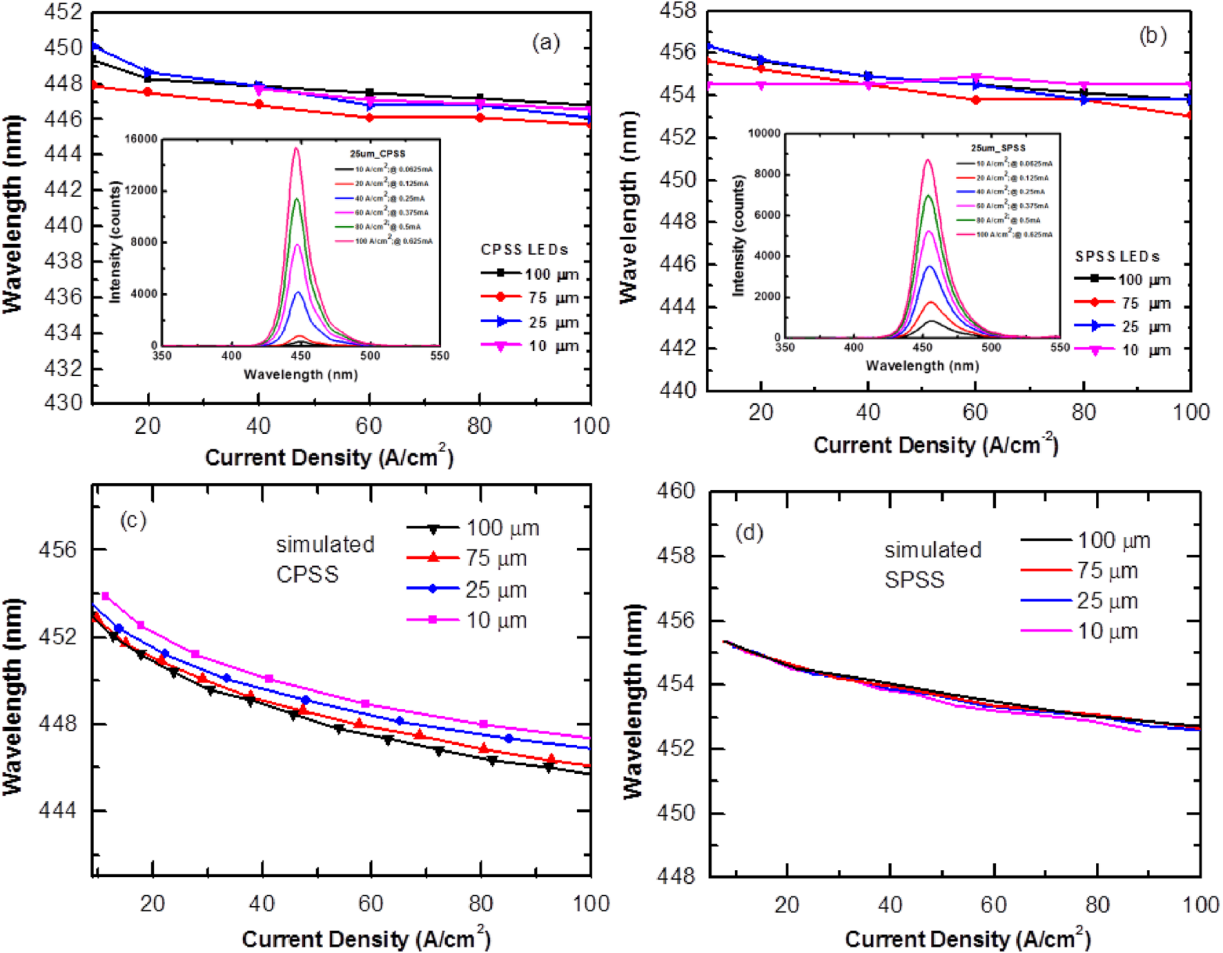
(**a**,**b**) are relationships between the wavelength (λp) and the current density with different chip sizes for CPSS and SPSS LEDs, respectively. (**c**,**d**) are simulation results of CPSS and SPSS LEDs, respectively. Insets of Figs. (**a**,**b**) show the typical spectra of LED(25) with CPSS and SPSS, respectively.

Figure 7a,b show the characteristics of the wavelength (λp) of the CPSS and SPSS LEDs as a function of the current density. The LED(100), LED(75), LED(25), and LED(10) with CPSS were blue-shifted by 2.52 nm, 2.16 nm, 4.05 nm, and 1.8 nm, respectively, when the current density increased from 10 to 100 A/cm^2^. In contrast, the LED(100), LED(75), LED(25) and LED(10) with SPSS were blue-shifted by 2.07 nm, 2.52 nm, 2.52 nm, and 0 nm, respectively, when the current density increased from 10 to 100 A/cm^2^. The typical spectra of LED(25) with CPSS and SPSS were shown in the insets of Fig. 7a,b, respectively. Obviously, the wavelength shift phenomenon for the SPSS LEDs was lower than that of the CPSS LEDs under the same device size. The simulation result shown in Fig. 7c,d further confirmed the trend. This phenomenon was due to the shielding effect caused by the electric field. For the CPSS LEDs, the polarization field was much stronger compared to the SPSS LEDs. This polarization caused the quantum confined stark effect (QCSE). When the injected current increased, an electric field formed due to the asymmetry of the electron–hole. This field was opposite to the polarized electric field built in MQW, which canceled the QCSE and caused the blue shift. Since the semi-polar epitaxial wafer had a much weaker polarization field, it could moderate the shielding effect and improve the problem of blue shift in device wavelengths.

## Conclusion

In this paper, the c-plane and (22$\overline{4}$3) pattern sapphire substrates were used to grow blue epitaxial wafers and make devices with light-emitting areas of 100 μm × 100 μm, 75 μm × 75 μm, 25 μm × 25 μm, and 10 μm × 10 μm. The different LED sizes of had a great influence on the electrical properties such as J–V curve. The small-sized LEDs had a larger current density under the same voltage and presented a smaller forward voltage. The electrical properties of the small-sized CPSS and SPSS LEDs were better than those of the large-size LEDs. Regarding the optical characteristics of LEDs, although the IQE of the smaller chip size could be worse due to the sidewall surface state, the LEE would have an inverse trend. Therefore, the output power density of the 25 $\mathrm{\mu m}$ chip provided better output power density in both cases. The larger devices exhibited maximum IQE at the lower current density compared to the smaller devices, and the IQE had a larger droop as the current density increased for the CPSS LEDs. In addition, the low blue-shift phenomenon of the SPSS LEDs was obtained as compared to that of the same size of CPSS LEDs.

## Methods

In this study, CPSS and SPSS blue epitaxial structures were grown by metalorganic chemical vapor deposition (MOCVD) in a low-pressure reactor. The semipolar (20$\overline{2}$1) GaN epilayer was grown on a (22$\overline{4}$3) PSS. The offset angle of the (22$\overline{4}$3) sapphire was adjusted to obtain a (20$\overline{2}$1)GaN surface exactly parallel to the surface of the substrate surface. The angle between (22$\overline{4}$3) sapphire and *c* plane sapphire is 74.64°, while the angle between (20$\overline{2}$1) GaN and *c*-plane GaN is 75.09°. Detailed (22$\overline{4}$3) PSS preparation and (20$\overline{2}$1) GaN growth mechanism has been described in our recently publication^22^. The blue CPSS epilayers consisted of 2 μm thick n-type GaN, a 300 nm thick active layer, a 400 nm thick p-type GaN, and a 200 nm thick indium tin oxide (ITO) layer. Correspondingly, the SPSS LED consisted of a 4 μm thick n-type GaN, a 50 nm thick active layer, a 300 nm thick p-type GaN, and a 200 nm thick ITO layer. The LEDs created on these two blue epitaxial structures had four different sizes: LED(100), LED(75), LED(25), and LED(10), which were all used to evaluate the influence of chip size and optical characteristics.

**Figure 8:**
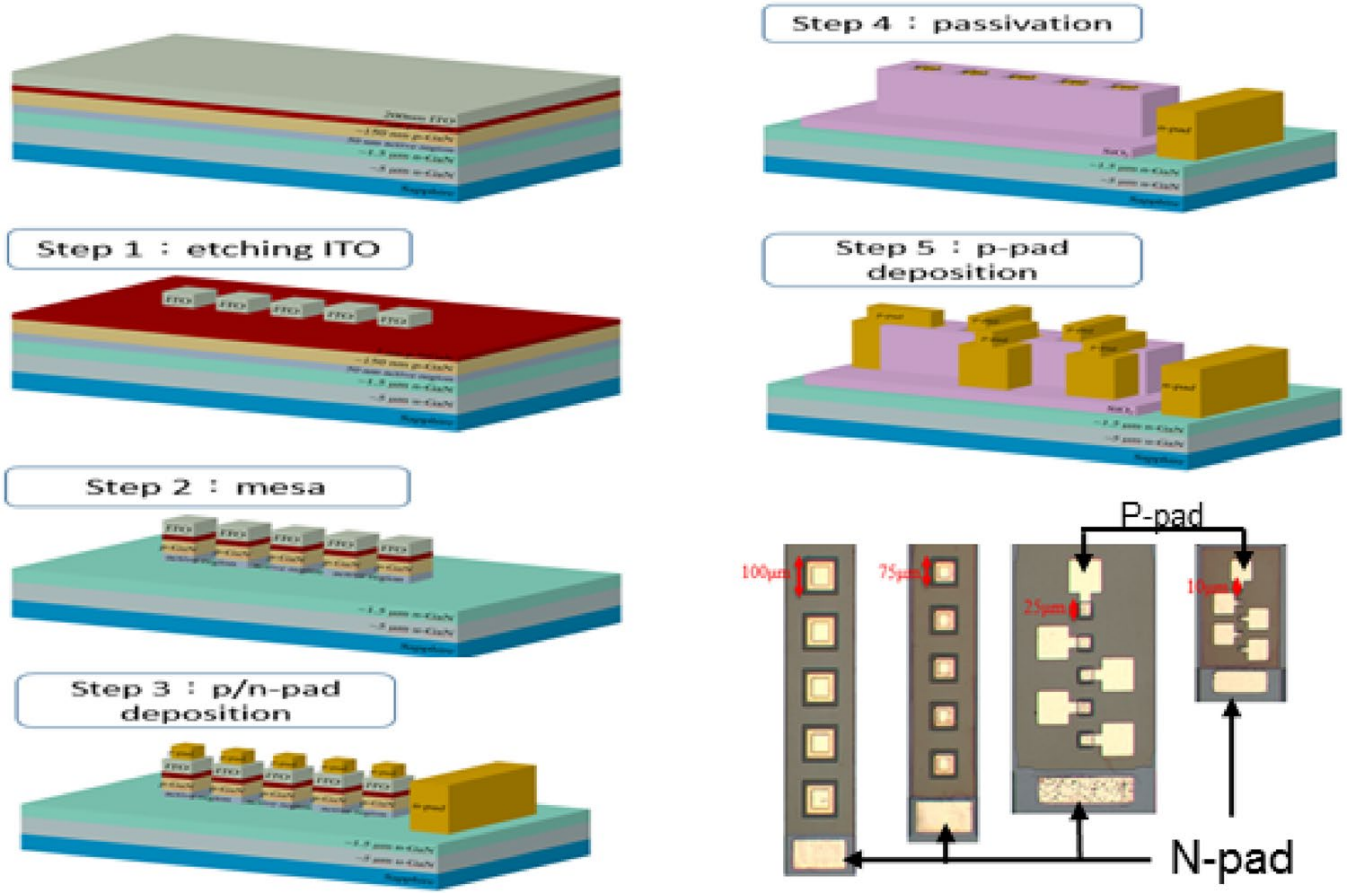
Process flowchart of μ-LEDs and OM image of blue LEDs after the whole process.

Figure 8 shows the complete process flow chart for the μ-LED fabrication. First, to define the light-emitting area on the epitaxial wafer, in step 1, wet etching the ITO and in step 2, dry etching the p-GaN and active layers process. Next, in step 3, n-pad and p-pad metals were deposited on n-GaN and ITO using an E-gun evaporation system. This was followed by depositing silicon dioxide for passivation and then opening the p-electrode area (step 4). Finally, the p-pad electrodes were deposited on the opening region, as shown in step 5 of Fig. 1. Optical microscope (OM) images of the blue LEDs with different size are also shown in Fig. 8. The LEDs could be successfully fabricated, even with sizes as small as 10 μm.

In order to form an Ohmic contact between the ITO layer and the p-GaN layer, it was necessary to anneal these samples at 525 °C for 10 min in the air before performing step 2. An E-gun evaporation system was used to deposit Ti/Al/Ti/Au on the ITO layer as a p-metal with thicknesses of 50 nm, 300 nm, 50 nm, and 60 nm respectively. In addition, Ti/Al/Ti/Au was deposited on the n-type GaN layer^23, 24^ with thicknesses of 50 nm, 1.8 μm, 50 nm, and 60 nm, respectively.

After the whole process for LED fabrication was completed, they were packaged by flip chip form. The electrical and optical characteristics of the device were measured using a multi-function power meter (Keithley 2400) and the integrating sphere detector (CAS 140B, Instrument Systems) in order to understand the influence of chip size on CPSS and SPSS blue LEDs. In this study, twenty devices were measured for each chip size.

## Simulation

To further explain the experimental results, 2D Poisson, drift–diffusion, and the Schrodinger solver (called 2D-DDCC) developed in NTU^25, 26^ were applied to model the performance of the μ-LEDs grown on CPSS and SPSS using different chip sizes. The solver could solve the Poisson, drift–diffusion, and localization landscape equations^26^ self-consistently. The parameters used were listed in Table 1. The simulation is majorly to tune for the same wavelength. So the c-plane and semipolar plane have different indium composition in the simulation. The QW number is also different due to the experimental devices. The SPSS structure does not have EBL so that EBL was not put in SPSS simulation. The n-GaN layer mobility for SPSS is smaller due to the observed larger sheet resistance is SPSS. In addition, the nonradiative lifetime in SPSS is also shorter in the bulk active region (MQW). This is due to the much lower EQE observed in SPSS structure. However, the IQE of SPSS LED did not exactly 10 times smaller than that of CPSS LED. Because there might be other thermal or LEE effect which did not considered in the simulation. After converging on the solution, we will need to solve Schrodinger equations to get the confined eigen states for EL calculation. Since carriers are only confined in the growth direction and are free in the lateral direction, the 1D Schrodinger solver was performed along the vertical y-direction at each lateral x-position to estimate the variation of the effective bandgap. The PL spectrum was calculated based on the Schrodinger solver. Due to the influence of the surface state at the sidewall, a higher concentration of defects with a shorter nonradiative lifetime (0.1 ns) were put on the sidewall within a 40 nm region from the sidewall on both sides. The exact position for one of the sidewall can be found in Fig. 5b from x = 100 μm. In addition, due to the higher contact resistance in the semipolar structure, the forward voltage of the simulated data was much smaller, as this effect was not included.

**Table 1:** Parameters used for simulation.

	CPSS	SPSS
MQWs	6 periods	3 periods
EBL	Yes	None
QW thickness	3 nm	3 nm
In composition in QW	15.5%	21.5%
Non-radiative lifetime in bulk active region (QW)	50 ns	10.0 ns
Non-radiative lifetime in sidewall	0.1 ns	0.1 ns
n-GaN layer ${\upmu }_{\mathrm{n}}$ (cm^2^/Vs)	100.0	50.0
p-GaN layer ${\upmu }_{\mathrm{p}}$ (cm^2^/Vs)	2.0	2.0
